# Diagnostic performance and clinical utility of metagenomic next-generation sequencing in suspected central nervous system infections: a prospective comparative study

**DOI:** 10.3389/fcimb.2025.1612628

**Published:** 2025-08-11

**Authors:** Xiao-guang Cao, Xiong-feng Zhu, Zha Yu, Chun-yan Wang, Min Shao, Hua-dong Meng, Chong-jian Huang

**Affiliations:** ^1^ Department of Emergency medical center, The First Affiliated Hospital of University of Science and Technology of China (Anhui Provincial Hospital), Hefei, Anhui, China; ^2^ Department of Emergency Medicine, The Third People’s Hospital of Hefei, Hefei, Anhui, China; ^3^ Intensive Care Unit (ICU), The Third Affiliated Hospital of Anhui Medical University (The First People’s Hospital of Hefei), Hefei, Anhui, China; ^4^ Department of Emergency Medicine, Suzhou Hospital of Anhui Medical University (Suzhou Municipal Hospital of Anhui Province), Suzhou, Anhui, China

**Keywords:** CNS infections, mNGS, CSF analysis, diagnostic accuracy, pathogen detection

## Abstract

**Objective:**

To assess the diagnostic performance and clinical utility of metagenomic next-generation sequencing (mNGS) in patients with suspected central nervous system (CNS) infections.

**Methods:**

prospective study was conducted from December 2019 to January 2024, enrolling 110 patients with suspected CNS infections. Cerebrospinal fluid (CSF) samples were subjected to mNGS, conventional biochemistry, and culture. Clinical features and outcomes were compared between patients confirmed with CNS infections and those without.

**Results:**

Of the enrolled patients, 69 were diagnosed with CNS infections. mNGS identified pathogens in 62 cases (77.11%), including 54 clinically confirmed true positives (49.09%), significantly surpassing traditional CSF culture (6.36%). mNGS reported results within 24 hours, considerably shorter than the 72~120 hours required for culture. Compared to the non-infection group, patients with CNS infections had significantly higher ICU admission(ICUA) rates, prolonged hospital stays, increased healthcare costs, and elevated rates of antibiotic adjustment and mNGS positivity (P<0.05). CSF turbidity, cell count, and protein levels were significantly elevated, while glucose and chloride levels were reduced. Logistic regression identified mNGS, CSF protein, and glucose levels as independent predictors of CNS infection. Receiver operating characteristic (ROC) analysis demonstrated superior diagnostic accuracy for continuous CSF variables over binary ones, with mNGS showing robust performance [area under the curve (AUC) = 0.794].

**Conclusion:**

mNGS offers rapid and accurate pathogen detection, outperforming conventional methods in sensitivity and turnaround time, and provides valuable guidance for individualized antimicrobial treatment in CNS infections.

## 1 Introduction

Sepsis is a common condition that is associated with unacceptably high mortality, according to the global burden of disease analysis, sepsis remains a life-threatening clinical syndrome with an alarming case-fatality rate of 26.7%, accounting for 19.7% of global mortality, predominantly driven by preventable infections in low-resource settings (Cecconi et al., 2018; Fleischmann-Struzek et al., 2020; Rudd et al., 2020). Although CNS infections account for a smaller proportion of sepsis cases, they are among its important causes. When CNS infections progress to sepsis or septic shock, patient prognosis worsens significantly, including increased mortality and longer hospital stays. These risks highlight the critical need for early and accurate pathogen detection in CNS infections (Hernandez Ortiz et al., 2018; Zhou et al., 2022). Conventional diagnostic methods, such as body fluid cultures and molecular assays, have limitations that frequently lead to diagnostic errors and delays (Filkins et al., 2020). mNGS, an emerging diagnostic tool, enables unbiased pathogen detection—including bacteria, viruses, fungi, and parasites—thus offering a rapid and accurate diagnostic approach. However, the current absence of large-scale, multicenter clinical trials results in heterogeneity of interpretation criteria and insufficient evidence-based support for routine clinical practice (Zhang et al., 2020). Consequently, this retrospective study analyzed suspected CNS infection cases in our province, aiming to evaluate the diagnostic value of mNGS in CNS infections and provide evidence to optimize the clinical management of these patients. To evaluate the diagnostic value of systemic infectious diseases and provide evidence for optimizing clinical management of these patients.

## 2 Materials and methods

### 2.1 Date collection

This study included 135 specimens collected between December 2019 to January 2024 in the Anhui Provincial Hospital. Ultimately, 110 patients were selected. All patient data were independently collected by two doctors and reviewed by a chief physician. The baseline data consist of age, gender, cerebrospinal fluid cell count, albumin, chloride, glucose levels, cerebrospinal fluid culture, and other pertinent indicators. This encompasses immediate mNGS examination(INE), antibiotic use at admission(AUA), empirical antimicrobial therapy(EAT), the accuracy of antimicrobial treatment(AAT), adjustments in the antimicrobial regimen(AAR), prognosis (recovery/worsening), ICUA, length of hospital stay(LHS), Hefei City medical insurance fees(HFMI), and hospitalization expenses(HE) extracted from the Donghua Electronic System.

### 2.2 Inclusion and exclusion criteria

**Figure 1:**
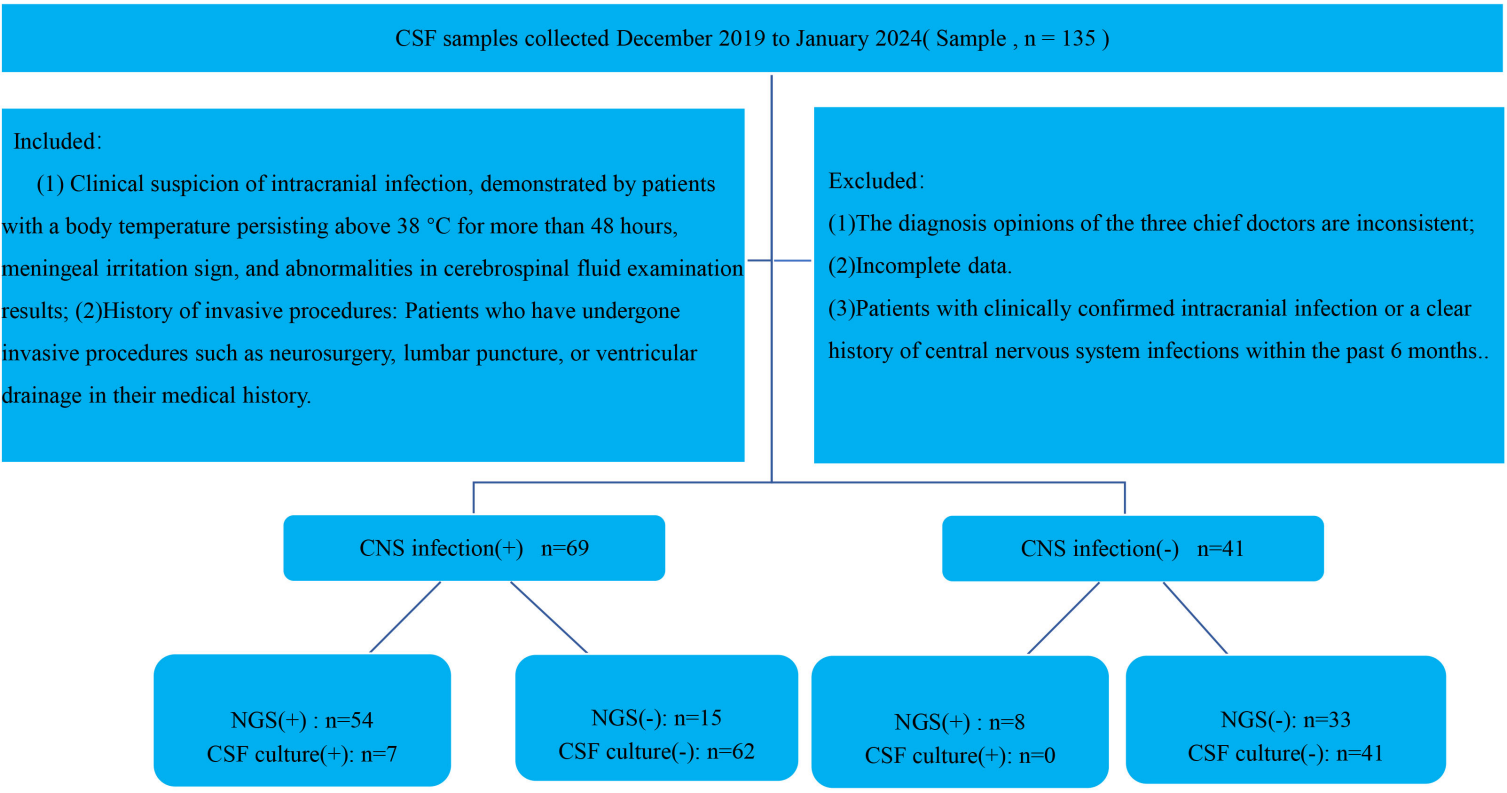
Flowchart illustrating patient enrollment, diagnostic grouping, and metagenomic next-generation sequencing (mNGS) results for suspected central nervous system (CNS) infections.

The inclusion criteria for this study are as follows:( (1) Clinical suspicion of intracranial infection, defined by a sustained body temperature above 38°C for more than 48 hours, the presence of meningeal signs or other suspicious clinical manifestations, and abnormal CSF test results (2); History of invasive procedures: Patients who have undergone invasive procedures such as neurosurgery, lumbar puncture, or ventricular drainage in their medical history. Exclusion criteria include (1): The diagnosis opinions of the three chief doctors are inconsistent (2); Incomplete data (3). Patients with clinically confirmed intracranial infection or a clear history of central nervous system infections within the past 6 months. All patients participating in the study, as well as their families, have been fully informed about the testing procedures and have provided informed consent (**Figure 1**).

### 2.3 Diagnostic criteria of CNS infection

Clinical Manifestations:1. The patient exhibits clinical signs and symptoms, including changes in consciousness and mental state, increased symptoms and signs of intracranial pressure, positive meningeal irritation signs, and focal symptoms resulting from intracranial inflammatory reactions. Systemic infection symptoms may include abnormal body temperature (>38°C or <36°C), increased white blood cell count, elevated heart rate, and altered breathing;2 (Tunkel et al., 2017 2017; McGill et al., 2016; Chinese Neurosurgical Society et al., 2021; Long et al., 2016). Imaging Findings: Inflammatory changes in brain tissue are observed on head CT and/or MRI scans, indicating the presence of intracranial inflammation;3. CSF Testing: Abnormalities in biochemical and routine CSF examinations include changes in appearance, elevated white blood cell counts (>10×10^6^/L), reduced glucose levels (<2.60 mmol/L), or a CSF-to-plasma glucose ratio of <2/3. Additionally, a CSF protein level exceeding 0.6 g/L is considered abnormal;4. For cases with atypical symptoms and signs, a comprehensive diagnosis is reached through the review and analysis of clinical data by three experts from ICU, neurosurgery, and neurology. The diagnosis takes into account the disease’s progression and laboratory examination results.

### 2.4 Research methods

#### 2.4.1 CSF detection

Routine and mNGS Testing for Intracranial Infections: The following procedures were performed for routine and mNGS testing: a total of 3~5 ml of CSF was collected via lumbar puncture. Various tests were subsequently conducted, including routine analysis, biochemical analysis, bacterial smear, and culture. For the culture, 3 ml of CSF was either directly sent for testing or inoculated into aerobic blood culture bottles, which were then transported to the laboratory within 2 hours at room temperature.

#### 2.4.2 DNA extraction, library preparation, and mNGS of CSF

1.5–3 ml CSF sample was collected from each patient by lumbar puncture in accordance with standard procedures. A 1.5 ml microcentrifuge tube with 0.6 ml sample and 250 μl, 0.5 mm glass bead were attached to a horizontal platform on a vortex mixer and agitated vigorously at 2800–3200 rpm for 30 min (O’Leary et al., 2016). Then 7.2 μl lysozyme was added for wall-breaking reaction. 0.3 ml sample was separated into a new 1.5 ml microcentrifuge tube and DNA was extracted using the TIANamp Micro DNA Kit (DP316, Tiangen Biotech) according to the manufacturer’ s recommendation. Nucleic acids RNA extracted from the clinical samples using the TIANamp Micro RNA Kit (DP431, Tiangen Biotech, Beijing, China) in accordance with the manufacturer’s standard protocols. The reverse transcription reaction was performed to generate single-strand cDNA, followed by the synthesis of double-strand cDNA using the PMseq™ RNA Infection Pathogen High-throughput Detection Kit according to the manufacturer’s instructions. Then, DNA libraries were constructed by enzymatic fragmentation for 20 minutes at 37°C, followed by end repair, adapter ligation, and PCR amplification using the PMseq™ RNA Infection Pathogen High-throughput Detection Kit (BGI-Shenzhen, China), according to the manufacturer’s instructions. Each library was uniquely barcoded to distinguish individual patient samples. The resulting libraries were purified using magnetic beads and assessed for quality using an Agilent 2100 Bioanalyzer. Quality-approved libraries were pooled in equimolar amounts, converted into DNA nanoballs (DNBs), and sequenced on the BGISEQ-50/MGISEQ-2000 platform (MGI Tech Co., Ltd, China). A negative control was included in each sequencing run to monitor for potential contamination. Sequencing runs typically included 10 to 20 samples per batch to ensure efficient throughput while minimizing the risk of cross-contamination.

#### 2.4.3 Interpretation of mNGS data

Raw sequence data were first filtered by removing common background microorganisms and low-quality reads. Then the filtered sequences were mapped to the human reference database (hg38) using Burrows-Wheeler alignment to computationally subtract the human sequence. The remaining data were further classified by simultaneously alignment to Pathogens Metagenomics Database (Refseq). The classification reference databases were downloaded from the National Center Biotechnology Information (NCBI) (ftp://ftp.ncbi.nlm.nih.gov/genomes/). RefSeq contains 4945 whole genome sequence of viral taxa, 6350 bacterial genomes or scaffolds, 1064 fungi and 234 parasites related to human infections (Li and Durbin, 2009; O’Leary et al., 2016; Miao et al., 2018).

#### 2.4.4 Criteria for a positive mNGS result

The criteria for positive results (Li and Durbin, 2009; Infectious Diseases and Cerebrospinal Fluid Cytology Group and Neurology Branch of Chinese Medical Association, 2021; Zhang S. et al., 2022) were as follows: 1) mNGS identified bacteria (mycobacteria and nocardia excluded), virus and parasites when the coverage rate was 10-fold greater than that of any other microorganisms. 2) mNGS identified Mycobacterium tuberculosis when the genus-specific read number ≥ 1. 3) mNGS identified nontuberculous mycobacteria and nocardia when the mapping read number (genus or species level) was in the top 10 in the bacteria list. 4) mNGS identified fungi when the coverage rate was 5-fold greater than that of any other microorganisms.

### 2.5 Statistical analysis methods

Data processing and analysis were performed using SPSS 22.0 statistical software. The analysis methods used were as follows: continuous variables(CV) with a normal distribution were presented as mean ± standard deviation (SD), while those with non-normal distribution were expressed as M (P25 and P75). Binary variables(BV) were reported as numbers (percentage). Variable comparisons were conducted using appropriate statistical tests, including the *t*-test, the non-parametric Mann–Whitney U test, or the *χ*2 test. The diagnostic accuracy of clinical features and laboratory characteristics was assessed and compared using the AUC of ROC. Data analysis was performed using SPSS 22.0 and Medcala 18.11.3 software. Statistical significance was set at P < 0.05. All Figures were created using the R programming language.

## 3 Result

### 3.1 Patient baseline characteristics

A total of 135 patients were enrolled, and 110 patients were ultimately included in the study, comprising 73males and 37 females. Based on the final diagnosis, patients were divided into two groups: 69 patients with CNS infections (observation group) and 41 patients without CNS infections (control group). Pathogens were identified in 7 patients through routine culture and other diagnostic methods, while mNGS detected pathogens in 62 patients.

In the CNS infection patients, the ICU, LHS, HE, AAR, mNGS positivity rate, and the proportion of CSF with cloudy appearance, as well as CSF cell count and albumin levels, were all significantly higher than those in non-CNS infection patients; In contrast, the prognosis improvement rate and CSF chloride and glucose levels were lower (P<0.05) (**Table 1**).

**Table 1:** Univariate analysis of factors associated with central nervous system infections.

Variables	CNS infection (n = 69)	Non-CNS infection (n = 41)	*Z/t/χ²*	*P*
Age	53.000 (33.000, 65.000)	52.000 (42.000, 64.000)	0.074	0.941
Gender(M/F)	49 (71.014)/20 (28.986)	24 (58.537)/17 (41.463)	1.794	0.180
mNGS(+/-)	54 (78.261)/15(21.739)	8 (19.512)/33 (80.488)	36.090	<0.001
INE(Y/N)	41 (59.420)/28 (40.580)	25 (60.976)/16 (39.024)	0.026	0.872
AUA(Y/N)	48 (69.565)/21 (30.435)	27 (65.854)/14 (34.146)	0.163	0.686
AAR(Y/N)	40 (57.971)/29 (42.029)	12 (29.268)/29 (70.732)	8.500	0.004
EAT(Y/N)	48 (69.565)/21 (30.435)	30 (73.171)/11 (26.829)	0.162	0.687
Prognosis(R/W)	41 (59.420)/28 (40.580)	33 (80.488)/8 (19.512)	5.185	0.023
ICA(Y/S)	49 (71.014)/20 (28.986)	16 (39.024)/25 (60.976)	0.888	<.0.001
HFCMIF(Y/N)	17 (24.638)/52 (75.362)	8 (19.512)/33 (80.488)	0.385	0.535
LHS	24.000 (13.000, 39.000)	13.000 (8.000, 19.000)	3.627	<0.001
HE(10.000yuan)	8.935 (2.494, 17.997)	3.533 (1.478, 6.479)	2.989	0.003
CSF				
turbidity(+/-)	42 (60.870)/27 (39.130)	11 (26.829)/30 (73.171)	11.936	<0.001
Cell-bv(+/-)	65 (94.203)/4 (5.797)	16 (39.024)/25 (60.976)	40.335	<0.001
Cell-cv	194.000 (45.000, 469.000)	7.000 (2.000, 15.000)	7.490	<0.001
Chloride-bv(+/-)	36 (52.174)/33 (47.826)	9 (21.951))/32 (78.049)	9.718	0.002
Chloride-cv	118.813 ± 6.537	123.771 ± 6.432	3.869	<0.001
Glucose-bv(+/-)	58 (82.857)/11 (15.942)	24 (58.537)/17 (41.46)	8.828	0.003
Glucose-cv	2.810 (1.910, 3.760)	4.010 (3.170, 4.770)	4.371	<0.001
Protein-bv(+/-)	61 (88.406)/8 (11.594)	23 (56.098)/18 (43.902)	14.873	<0.001
Protein-cv(+/-)	1.310 (0.803, 2.463)	0.560 (0.422, 1.022)	4.592	<0.001

‘+’/’–’ indicate positive/negative; ‘Y’/’N’ indicate yes/no; ‘R’/’W’ indicate recovery/worsening; ‘M’/’F’ indicate male/female. INE, immediate mNGS examination; AUA, antibiotic use at admission; EAT, empirical antimicrobial therapy; AAT, accuracy of antimicrobial treatment; AAR, adjustments in the antimicrobial regimen; Prognosis, prognosis (R = recovery, W = worsening); ICUA, ICU admission; LHS, length of hospital stay; HFMI, Hefei City medical insurance fees; HE, hospitalization expenses; CNS, central nervous system; CSF, cerebrospinal fluid.

### 3.2 Correlation and concordance between mNGS and CSF culture

**Figure 2:**
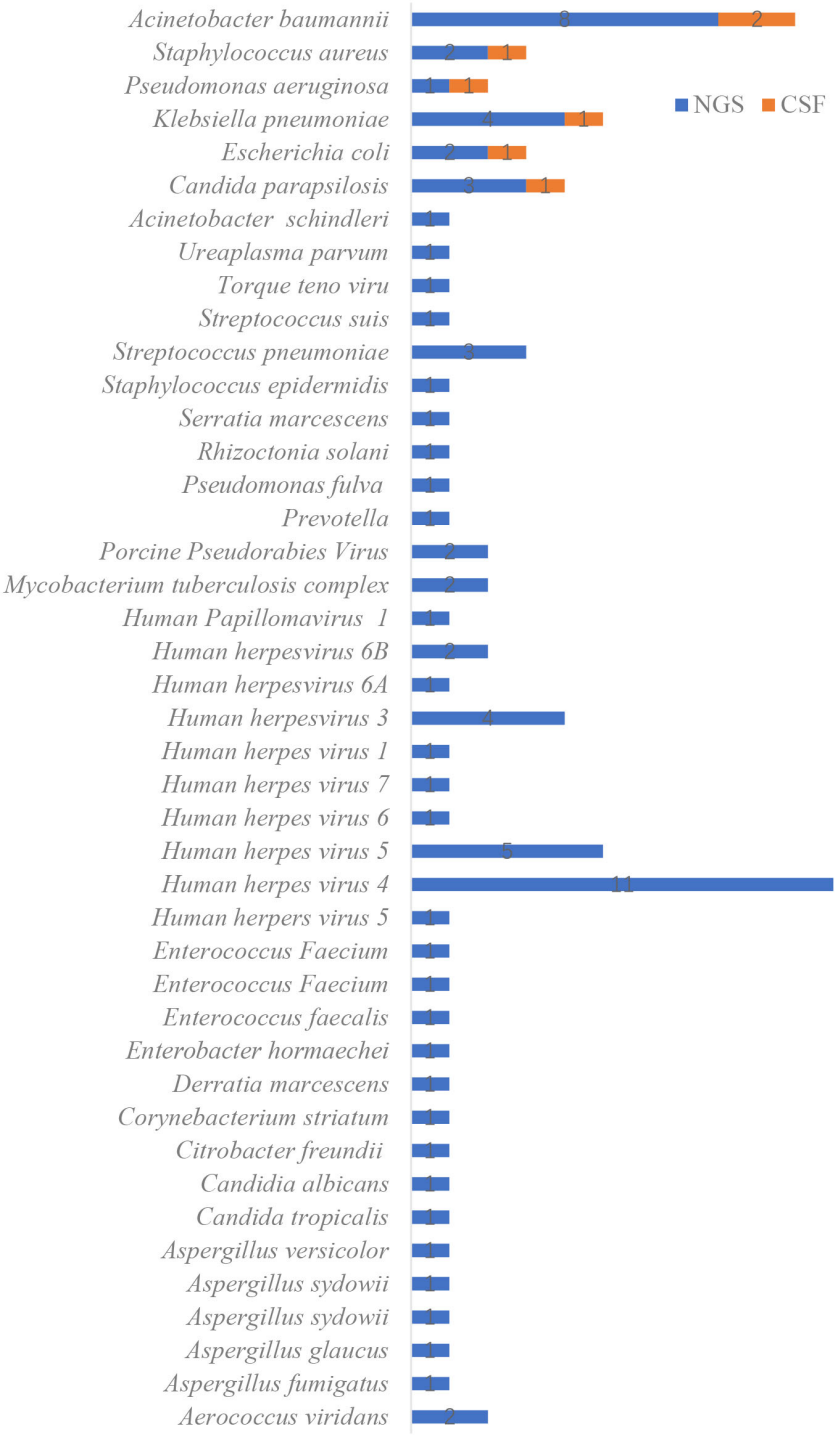
Distribution of pathogens identified by metagenomic next-generation sequencing (mNGS) compared with conventional cerebrospinal fluid (CSF) culture methods.

Among the 110 CSF culture samples, only 7 were positive (6.36%), including Klebsiella pneumoniae (1 case), Acinetobacter baumannii (2 cases), Escherichia coli (1 case), *Staphylococcus aureus* (1 case), Pseudomonas aeruginosa (1 case), and Candida parapsilosis (1 case). No sample cultured multiple pathogens. In contrast, mNGS identified 80 pathogens in 62 positive samples, with 6 samples containing two or more pathogens. The most common pathogens identified were Klebsiella pneumoniae (4 cases), Acinetobacter baumannii (8 cases), Human herpesvirus 5 (4 cases), and Human herpesvirus 4 (11 cases) (see **Figure 2**). Furthermore, the average turnaround time for pathogen identification by mNGS was approximately 24 hours, whereas the CSF culture had a turnaround time of 72 to 120 hours.

### 3.3 Multifactor logistic regression analysis

**Figure 3:**
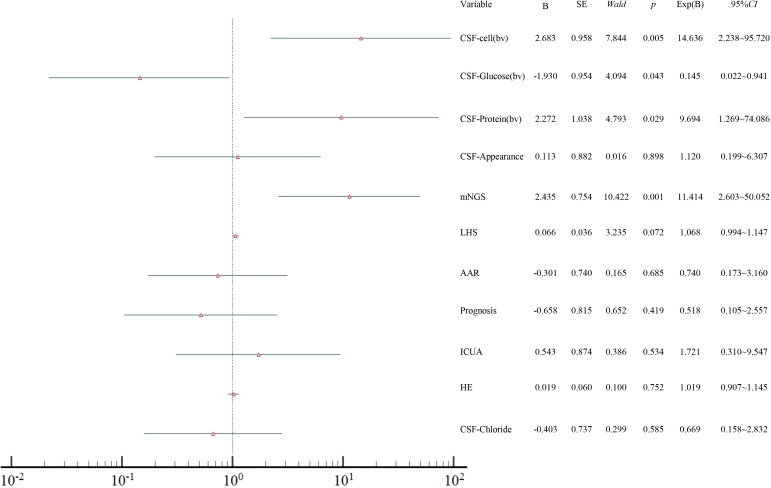
Forest plot of multivariate logistic regression analysis showing independent factors associated with CNS infection. AAR, adjustments in the antimicrobial regimen; ICUA, ICU admission; LHS, length of hospital stay; HE, hospitalization expenses; CSF, cerebrospinal fluid; bv, binary variables.

Logistic regression analysis indicated that mNGS was independently associated with CSF protein, glucose levels, and CNS infections (P<0.05) (**Figure 3**).

### 3.4 Analysis of diagnostic-effectiveness of different indexed

**Figure 4:**
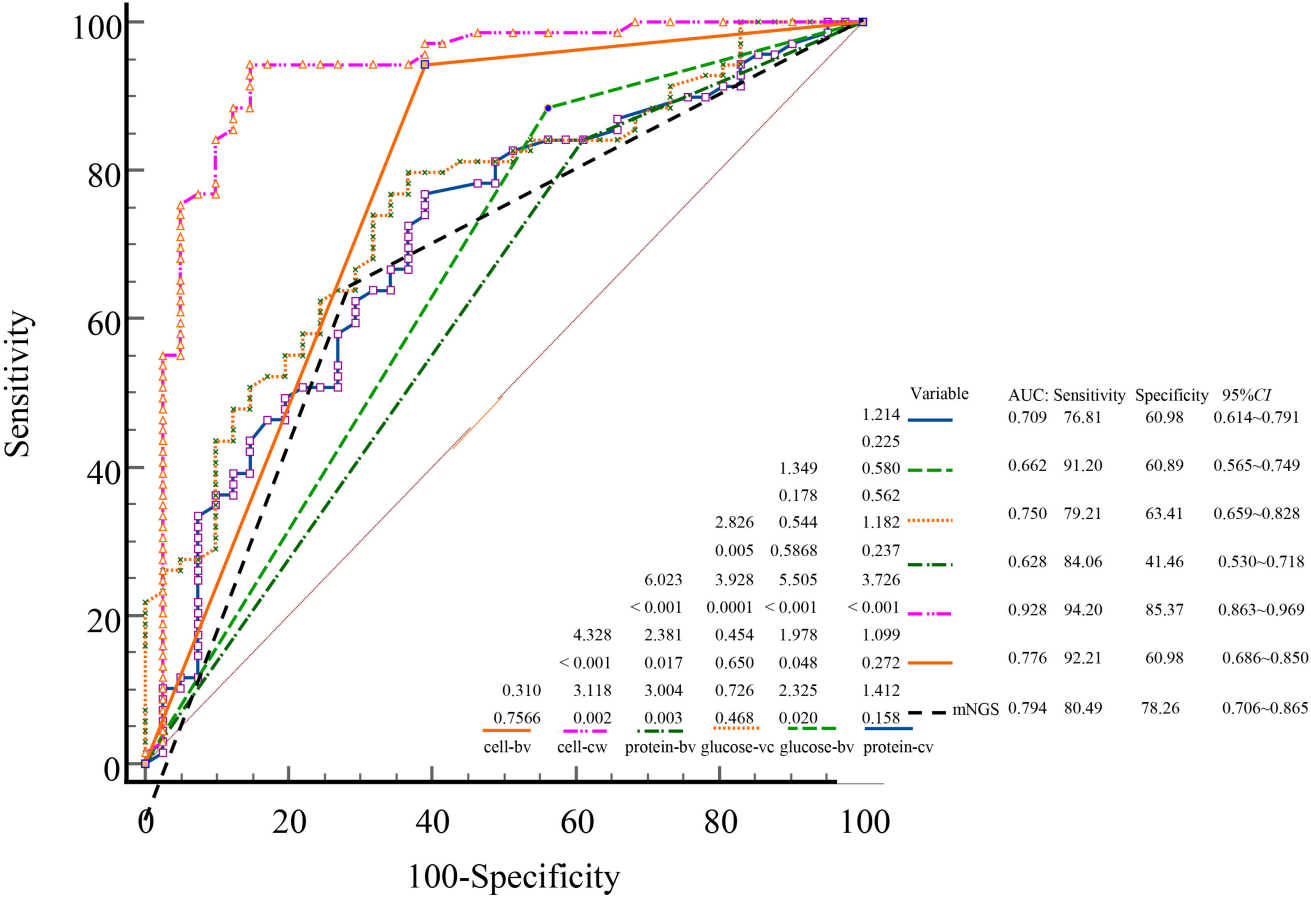
ROC curves comparing the diagnostic performance of mNGS and conventional CSF indicators for CNS infections. ROC, receiver operating characteristic; AUC, area under the curve; mNGS, metagenomic next-generation sequencing; CSF, cerebrospinal fluid; CNS, central nervous system; cv, continuous variables; bv, binary variables.

Using the final clinical diagnosis of CNS infection as the reference standard, ROC curves were plotted for mNGS and CSF parameters, including cell count, glucose, and protein. Each variable was assessed in both binary (bv) and continuous (cv) forms (**Figure 4**). The AUC for mNGS and for CSF cell count, glucose, and protein were 0.794; 0.776 (bv)/0.982 (cv) (P = 0.225, Z = 1.214); 0.628 (bv)/0.750 (cv) (P = 0.005, Z = 2.826); and 0.662 (bv)/0.709 (cv) (P < 0.001, Z = 4.328), respectively (**Figure 4**). Continuous cell count demonstrated the highest diagnostic accuracy; additionally, when treated as binary variables, both mNGS and CSF cell count showed significantly better diagnostic performance than CSF glucose and protein (P < 0.05).

## 4 Discussion

CNS infections are a common cause of neurological disease and remain a major clinical challenge (Simner et al., 2018; GBD 2016 Meningitis Collaborators, 2018). Due to limitations in current diagnostic approaches patients often experience delayed treatment, prolonged hospitalization, increased healthcare costs, and even death (Brouwer et al., 2012; Acuña et al., 2022). In our study, patients with CNS infections had significantly longer hospital stays, higher ICU admission rates, and greater medical expenses compared to those without infection (P < 0.05). These differences may be attributed to the nonspecific clinical presentation of CNS infections, delays in etiological diagnosis, and reliance on empiric therapy in the absence of rapid confirmatory testing. These findings further highlight the urgent need for timely and accurate diagnostic strategies to improve clinical outcomes (Posadas and Fisher, 2018; Andrade et al., 2024).

Routine CSF biochemical analysis remains the primary diagnostic method due to its efficiency and cost-effectiveness (Hrishi and Sethuraman, 2019; Singh, 2024). Inflammatory responses from infections alter CSF parameters, including abnormal appearance, glucose, protein levels, and elevated white cell count, making these indicators key references in diagnosing CNS infections (Subspecialty Group of Neurology tSoPCMA, 2019; Zhang et al., 2022). This aligns with our findings, where CNS infection patients showed significantly higher CSF white cell counts, protein levels, and turbidity, and lower glucose levels than non-infection groups (P < 0.05). Logistic regression analysis indicated these parameters independently correlated with CNS infections. Additionally, we observed lower chloride levels in CNS infection patients, potentially due to pathogen-related chloride homeostasis disturbances (Wen et al., 2022), enterovirus-induced diarrhea (Koopmans et al., 2023), antidiuretic hormone dysregulation, or diuretic use (Johanson et al., 1992; Kumar et al., 2021).

In current clinical practice, laboratory indicators are often dichotomized as “normal” or “abnormal,” which may lead to loss of diagnostic detail and clinical nuance (Altman and Royston, 2006; Shinkins and Perera, 2013). In this study, CSF cell count analyzed as a continuous variable yielded a markedly higher AUC (0.982) than its binary counterpart (0.776). Similar patterns were observed for glucose (0.750 vs. 0.628) and protein (0.709 vs. 0.662). These results indicate that continuous variables retain more diagnostic information, allow finer resolution of disease severity, and improve the sensitivity and precision of ROC-based analysis. This highlights the importance of preserving quantitative data in clinical diagnostics rather than relying on simplified threshold-based classification. Future multicenter, prospective studies are warranted to validate these observations.

Although routine CSF biochemical analysis remains a useful tool in the initial assessment of CNS infections, its lack of pathogen specificity limits its diagnostic value. CSF culture, while considered the diagnostic gold standard, suffers from low sensitivity (Mengistu et al., 2013); in our study, only 6.36% (7/110) of cultures were positive, each identifying a single pathogen. This low yield may result from sampling challenges, prior antibiotic use, or low microbial load (Calderaro et al., 2016; Pope et al., 2023; Su et al., 2024; Wang et al., 2024). In contrast, mNGS is less affected by these limitations and has emerged as a valuable diagnostic tool owing to its high throughput, broad-spectrum pathogen detection, and rapid turnaround time (Wilson et al., 2019; Lin et al., 2024; Streithorst et al., 2024). In this study, mNGS achieved a significantly higher overall detection rate (77.11%), with 54 cases confirmed as true positives (49.09%), markedly outperforming conventional diagnostic methods. The mNGS positivity rate and the proportion of patients requiring antibiotic adjustment were both significantly higher in the CNS-infected group compared to the non-CNS group (P < 0.05), underscoring the clinical value of mNGS in distinguishing infectious from non-infectious etiologies and in guiding individualized antimicrobial therapy. ROC curve analysis further confirmed the diagnostic performance of mNGS, yielding an AUC of 0.794—second only to CSF cell count analyzed as a continuous variable. This result is consistent with previous reports, which documented AUC values for mNGS ranging from 0.659 to 0.846 depending on pathogen type (Xing et al., 2020; Yuan et al., 2024). Notably, the positivity criteria used in our study were based on internationally and domestically recognized expert consensus (Li and Durbin, 2009; O’Leary et al., 2016; Miao et al., 2018), which has been widely endorsed by clinicians in China, ensuring consistency in interpretation and methodological comparability. Collectively, these findings highlight the importance of mNGS in achieving rapid and accurate pathogen identification, particularly in patients with complex CNS infections.

Despite its high diagnostic yield, mNGS is not without limitations. In our cohort, 15 patients were clinically diagnosed with CNS infection despite negative mNGS results. These patients exhibited typical features of CNS infection—such as fever, altered mental status, meningeal signs, CSF pleocytosis, and hypoglycorrhachia—and showed clinical improvement following empirical antimicrobial therapy. These false-negative findings may be attributed to low pathogen burden, atypical organisms, prior antibiotic exposure, or limitations in sequencing depth and bioinformatic filtering. Conversely, 8 patients in the non-CNS group yielded low-abundance mNGS-positive results for background or latent organisms such as *Torque teno virus* and *Human herpesvirus 4 virus*. None of these patients had clinical symptoms or laboratory evidence suggestive of CNS infection, and all were ultimately diagnosed with non-infectious etiologies. These cases underscore the necessity of interpreting mNGS results in conjunction with clinical context to avoid overdiagnosis due to contaminants or colonizing organisms. Viral CNS infections further complicate the diagnostic landscape. Conventional methods such as PCR and serology are highly pathogen-specific and prone to false negatives or missed diagnoses (Agut et al., 2016; Khan et al., 2024; Marvik and Dudman, 2024). In clinical practice, CSF volume limitations often restrict parallel molecular testing. Unlike these targeted assays, mNGS offers broad-spectrum detection without prior assumptions, enabling the identification of unexpected or coexisting viral pathogens, including mixed infections.

Taken together, mNGS should be regarded as a powerful adjunct rather than a stand-alone tool in the diagnostic workup of CNS infections, especially in culture-negative or diagnostically uncertain cases where conventional methods fall short. However, the interpretation of negative or low-abundance results requires careful clinical correlation. Therefore, further prospective, multicenter studies are warranted to refine mNGS-based pathogen detection strategies and to guide individualized antimicrobial and antiviral decision-making.

### 4.1 Limitations

First, the single-center design and relatively modest sample size may limit the generalizability of our findings. Larger, multicenter studies are warranted to validate the diagnostic performance and clinical utility of mNGS across diverse patient populations. Second, due to the retrospective nature of data extraction from the hospital information system, certain clinical variables—such as detailed antibiotic history, time interval between symptom onset and sampling, and severity scoring—were inconsistently recorded or unavailable, potentially affecting the interpretation of mNGS results. Third, although mNGS demonstrated a higher detection rate compared to conventional methods, this study lacked comprehensive comparison with pathogen-targeted PCR and other molecular diagnostic tools, which may provide additional diagnostic context for clinical decision-making. Fourth, limited CSF volume precluded complete virological serology and parallel validation testing in some cases, particularly for patients with suspected viral or mixed infections. Finally, this study primarily focused on the diagnostic value of mNGS; future investigations should evaluate the impact of mNGS-guided antimicrobial therapy on clinical outcomes and cost-effectiveness in patients with central nervous system infections.

## Data Availability

The datasets presented in this study can be found in online repositories. The names of the repository/repositories and accession number(s) can be found in the article/supplementary material.
